# Scuba Diving as a Form of Rehabilitation for People with Physical Disabilities

**DOI:** 10.3390/ijerph18115678

**Published:** 2021-05-26

**Authors:** Gabriela Henrykowska, Joanna Soin, Piotr Siermontowski

**Affiliations:** 1 Department of Epidemiology and Public Health, Medical University of Lodz, 90-752 Lodz, Poland; 2 Institute of Health Sciences, Stefan Batory State University, 96-100 Skierniewice, Poland; jsoin@pusb.pl; 3 Department Underwater Work Technology, Polish Naval Academy of the Heroes of Westerplatte, 81-103 Gdynia, Poland; nurdok@o2.pl

**Keywords:** scuba diving, rehabilitation in disability, quality of life

## Abstract

(1) Background: The exploration of the potential therapeutic benefits of scuba diving for the mental and physical health of people with physical disabilities. (2) Methods: The research was conducted on a group of 240 people (men and women) with physical disabilities, using the survey designed by one of the authors. The subjective sense of physical and mental fitness was analyzed in retrospective and real terms. (3) Results: Significant increases in self-esteem, belief in our own abilities (self-confidence) and improvement in the ability to engage in social interactions were observed in the group of scuba divers with disabilities compared to individuals with disabilities not practicing diving. The respondents also declared an improvement in the efficiency of the respiratory system and stressed that a water environment increased their motor skills and relieved pain. (4) Conclusions: Diving can become one of the forms of rehabilitation for people with disabilities. There is a need for further research to expand our understanding of the benefits and possible health problems involved in diving. These activities have a huge impact on improving the quality of life of people with disabilities.

## 1. Introduction

Disabilities are an ancient concept that have existed for as long as people have existed. A disability is often viewed as an unending burden, and people with disabilities have often been viewed as victims of circumstances, tragic figures whom society should pity. Despite great progress in medicine, the prevalence of disabilities in communities all over the world is still significant. In Poland, various types of disabilities affect 4–7 million people; over 3 million of them hold disability certificates [1].

The difficulties faced by people with disabilities are multiple, considering aspects starting from biological defects and deficits in the locomotor system through the mental sphere and the acceptance of their status to social aspects of the functioning of the disabled in a community and staying open to the surrounding world. The manner in which an individual affected by an illness or a disability deals with everyday life is different for each person. What influences the attempt to adapt oneself to a new situation is not only the degree and type of disability but, also, personality, family background or the financial situation of the individual concerned. Due to the multiplicity of factors that come into play, it is difficult to predict the time and manner in which individuals adapt to a new situation [2]. What is well-known, however, is the fact that proper and comprehensive rehabilitation plays a key role in the improvement of one’s condition and adaptation to disability.

Comprehensibility, referred to above, must be reflected in proper medical care, including continuous and personalized medical rehabilitation, as well as in the support granted by state institutions and organizations for individuals with similar problems. In addition, it is also important to educate society in order to reduce the barriers resulting from the lack of awareness and knowledge of disability-related issues.

Quite recently, diving has become a fashionable, prestigious and very interesting spare time activity [3,4]. Diving may be treated as a multidimensional therapy in water [5,6,7,8], which allows people with and without disabilities to share common activities [9]. Such rehabilitation involves comprehensive stimulation of the human body by social interactions through participation in classes, as well as by influencing the mental and physical spheres. Classes taking place in water and diving help to break the barriers resulting from being in a new environment, i.e., water, to regain the awareness of one’s own body and movements that one can make, feel the position of one’s own body, feel the possibility to give directions to movements and benefit from being able to increase independence in water [10,11].

The aquatic environment enables the relaxation of muscles, which, as a consequence, may have a positive effect on spasticity [12] and increase the joint range of motion often significantly limited in the natural environment [13,14]. In short, it enables improve movement and coordination in a way that is much easier than in a terrestrial environment. Such exercises have a positive effect on the respiratory system and blood circulation as a result of learning how to breathe correctly [15,16]. An individual who takes up diving has got an opportunity to learn and demonstrate independence by taking care of her/his own safety, as well as the safety of her/his companions. Diving encourages you to spend time with other people and, thus, gives an opportunity to be in a group whose members are people without disabilities, as well as people with locomotor system dysfunctions, giving the sense of affiliation with a group and responsibility for its members [17]. Diving may eliminate the limitations resulting from disabilities [9,12,18]; it often enables people with disabilities to exceed the possibilities of their able-bodied peers who report average levels of physical activity. The awareness of being a disabled person doing something unusual helps the disabled feel exceptional, which facilitates the process of accepting one’s own status [17,18,19,20]. However, one should not forget the possible hazards and risks entailed in scuba diving. Apart from the initial health condition, the factors having influence on human body include pressure under water, breathing gas, temperature, type of diving environment, mental barriers and possible problems with the equipment [21,22,23].

People with and without disabilities must obey the rules applicable to diving, be aware of hazards and strive to overcome problems connected to this kind of activity [17,23]. In addition, people with disabilities must consider their own dysfunctions, which may impact their preparations for diving and moving underwater. Furthermore, such dysfunctions may make the use of the scuba diving equipment more difficult or affect communication. What may help in such situations is the modification of the essential equipment and its adaptation to the needs of each diver with disabilities. People helping or assisting in diving may also provide valuable support [24]. They may be responsible for supervising the equipment of disabled individuals and for all the actions that they cannot perform. To communicate with the assistants, a certain method of communication needs to be established and then modified, if necessary, and adapted to one’s requirements [17,18,19,20].

Considering the above, the purpose of this study was to learn about the potential therapeutic benefits of diving for physical and mental health of the disabled. This findings are based on the analysis of the opinions submitted by the disabled themselves.

## 2. Materials and Methods

This study was carried out on a sample of 240 individuals (male and female) with physical disabilities. It was based on an original questionnaire (survey), which contained a set of closed-ended, semi-open and open-ended questions created using Google Forms. The survey was in Polish, since it was dedicated to Polish disables. The questionnaire was distributed online, using one of social networking sites (Facebook) among members of thematic groups, bringing together people with disabilities. Names of the groups in Polish were: “Niepelnosprawni”; “Kreatywni niepelnosprawni”; “Niepelnosprawni to tez ludzie”; “Sprawni niepelnosprawni”; “Niepelnosprawni sa wsrod nas”; “Niepelnosprawni podrozuja” and “Niepelnosprawni aktywni razem” (translating from Polish: “Disabled”, “Creative disabled”, “Disabled are also human beings”, “Disablesd are with us”, “Abled disabled”, “Traveling disabled” and “Disabled active together”). The survey was not addressed to divers or disabled divers. An invitation to take part in the survey and a link to the questionnaire were posted upon prior consent of administrators of the selected thematic groups. The research tool ensured anonymity to the respondents, and participation in the study was voluntary. From the call for participation (invitation), interested individuals could learn that the participation in the survey was voluntary, anonymous and each completed questionnaire was identified by a number. We also informed that the survey concerned the impact of scuba diving on physical and mental health. Moreover, it was explained that the goal of the survey was to expand the body of knowledge about scuba diving as a form of rehabilitation and that the survey target were people aged 18+ with physical disabilities. There was also a piece of information concerning the approximate time needed to fill out the questionnaire. Potential respondents were also informed that they may decide not to answer any specific question or stop participating at any time without any consequences. A reservation was made with regard to the risk of infringing the confidentiality of the data when transferring them over the internet (despite data confidentiality and anonymity being strictly observed by researchers) that went beyond the control of the survey team. It was also stressed that the filling out of the questionnaire was the equivalent of giving one’s consent to participate in the study.

Due to the anonymization of the questionnaire, the consent of the Bioethics Committee was not required, but for our special request, the decision of The Committee of Research Ethics #4/21 operating at the Naval Academy of the Heroes of Westerplatte was obtained. 

This research was the first part and first step in an international survey on scuba diving benefits, difficulties and fears experienced by people with disabilities.

The respondents were divided into two groups. Group A was composed of people with disabilities who had never participated in diving classes before the study. Group B included people with disabilities who had practiced diving.

Apart from providing sociodemographic and anthropometric data (weight and body mass), respondents from both groups were asked to assess the possible therapeutic benefits of scuba diving to people with disabilities.

For the survey, a five-point Likert rating scale was used [25], where 1 meant “strongly disagree” with the statement concerned, and 5 meant “strongly agree”.

The respondents were asked to express their opinions as to whether scuba diving could improve… (group A)/has improved… (group B) respondent’s:

- mental condition;
- self-assessment *;
- belief in your own abilities (self-confidence);
- network and quality of social interactions;
- overall physical fitness;
- muscle strength;
- success rate in acquiring new motor skills;
- respiratory system efficiency;
- comfort by relieving pain.

* Self-assessment is the process of looking at oneself—in particular, at one’s actions, attitudes and capabilities or other socially relevant characteristics. It can be positive or negative. This concept should not be considered as the same as self-esteem, which, according to the Oxford Language Dictionary, means being confident in one’s own worth; self-respect.

The obtained data were subject to a Shapiro–Wilk test, Mann–Whitney *U* test and a descriptive statistical analysis.

## 3. Results

The questionnaires were filled in by 240 individuals diagnosed with a disability. For formal reasons, the answers of 58 respondents were rejected. Further analyses covered only the questionnaires that were fully completed. Questionnaires filled out by respondents whose declared age was under 18 were rejected, together with questionnaires in which the respondents did not provide answers about the type of disability, its severeness or simply failed to answer all questions. Hence, further investigations focused on 182 correctly completed questionnaires (group A: *n* = 118; group B: *n* = 64).

The analysis of the examined group is presented in Table 1. Men represented two-thirds of the analyzed group (65.9%). The youngest respondent was 22 and the oldest one 75 years old.

In the examined group, dysfunctions of the musculoskeletal system were the reasons for disability in 78% of respondents (142 people); 4.4% of the respondents stated that their disability concerned vision dysfunctions, and the remaining respondents suffered from neurological diseases.

Nearly half of the respondents (89 people, 48.9%) held a certificate of disability stating that their condition was severe, while 34 respondents (18.7%) were diagnosed with a mild degree of disability.

The Polish law provides for three degrees of disability: severe, moderate and mild.

A person with severe disability is a person with an impaired ability of the organism, who is incapable of working or capable of working only in a protected working environment who, in order to fulfil her/his social roles, requires permanent or long-term care and the assistance of other people, because s/he is unable to lead an independent life.

A person with moderate disability is considered to be a person with a physical impairment who is unable to work or able to work only in a protected working environment or who requires temporary or partial assistance from others in order to fulfil their social roles.

A person with a mild degree of disability is a person with a physical impairment of the body such that significantly reduces the ability to perform work in comparison with the ability of a person with similar occupational qualifications and full psychological and physical capacity or who has limitations in fulfilling social roles that can be compensated with the use of equipment such as orthopaedic appliances, aids or technical devices.

The disabilities of almost one-third (1/3) of the respondents (*n* = 51) were congenital. Nearly 40% of the respondents became disabled as a result of a disease (*n* = 71); in 60 people, their disability was the result of an injury.

Among the individuals with acquired disabilities, the largest group was made of people who were disabled for 6 to 10 years (90 people). Every fifth respondent was a disabled person for between a year and 5 years; the remaining respondents lived with their disability for over 10 years.

One hundred and eight people (59.3% of the respondents) had an opportunity to take part in rehabilitation classes conducted in an aquatic environment; 64 individuals, i.e., 35.17% of all respondents, took part in scuba diving classes.

Following the adopted research methods, each group of respondents (A—non-divers, *n* = 118; B—divers, *n* = 64) was asked to assess certain aspects of scuba diving.

Respondents in both groups (divers and non-divers) stated that diving could impact their mental condition. Although 11.02% of the respondents marked a neutral point on the scale and five people had a negative opinion, the median was 5 (in both groups). It could be evidence of a very positive impact of diving on the mental health of the respondents (Table 2).

Similar distributions of answers were observed for answers to the question about the impact of diving on the improvement of self-esteem. Only 4.24% of respondents from group A (non-divers) had a negative opinion. The median response in both groups was 5. The analysis of the response distributions demonstrated that as many as 71.87% of the group B respondents ticked the highest score of the scale (Table 3).

Another examined parameter was the impact of diving on the improvement of belief in one’s own abilities. Again, divers with disabilities gave scores of 4 and 5. Despite a wider distribution of responses in the group of non-divers, the vast majority of respondents (88.14%) expected their self-confidence to increase after diving classes. The median in both groups was 5 (Table 4).

When analyzing the distribution of the answers concerning the impact of diving on the improvement of social interactions, it was noticed that non-divers responded similarly as in the case of previous questions. Skeptics accounted for only 5.08% of this group. A Similar number of respondents in both groups chose a neutral answer. Additionally, most of the respondents in both groups (A—81.36% and B—82.81%) indicated that diving might improve the scope and quality of their social interactions (Table 5).

Respondents’ answers to the question about the effect of diving on the improvement of physical fitness were quite varied, although the median in both groups was 4. Almost 40% of non-divers expressed high hopes that diving could improve their physical fitness. However, the incidence of the highest score (5) in the diving group was below 30% (Table 6).

Significantly diverse responses were given for the impact of diving on the increase in muscle strength. The median in both groups was 4. A neutral answer on the Likert scale was chosen by as many as 43.75% of divers and 30.51% of non-divers. A negative opinion was expressed by over 17% of the respondents in group A and only 6.25% in group B. However, the opinions of over 50% of the respondents were positive (Table 7).

All the respondents from group B (divers) indicated that diving broadened their motor skills. Among the people who have not dived yet, as many as 88.13% expected that this physical activity would bring the indicated benefits. Only four people expressed a negative opinion. The median in both groups was 5 (Table 8).

Every fourth respondent from group A and every third respondent from group B did not have a clear opinion on the impact of diving on the efficiency of the respiratory system. However, more than half of the respondents indicated significant benefits of diving. The total number of 4- and 5-score responses in both groups was around 70%. This was also confirmed by the median, which, in both groups, was 5 (Table 9).

The respondents were divided over the question concerning the impact of diving on pain relief. Over 70% of the respondents in both groups indicated that they rather agree with the proposed statement. This was confirmed by the median, which was 4 in both cases. The neutral position was marked by approx. 10% of the respondents in the non-diving group and 20% in the group of divers (Table 10).

The analysis of the answers provided by the respondents demonstrated that the lowest marks (2 and 1) were given very rarely. In all the assessed parameters, scores 5 and 4 (the highest) prevailed. The observed differences between non-divers and divers in categories 4 and 5 of the Likert scale were statistically significant in the respondents’ opinions on the impact on their mental conditions (*p* = 0.0137), self-esteem (*p* = 0.0145) and on their belief in their own abilities (*p* = 0.0093). In the other six categories, no statistically significant differences were observed. On the five-point Likert scale, medians of 4 to 5 indicated a very positive assessment of diving as a possible form of rehabilitation.

## 4. Discussion

People with disabilities are constantly seeking to find methods and forms of rehabilitation that would improve their everyday life functions. This study was intended to find out what physically disabled people (independently of the nature of their disabilities) think of the potential impact of scuba diving on their health at three levels: physical, mental and social. Therefore, we observed how respondents assessed the impact of scuba diving on all the listed areas. Such a holistic (like in our study) approach to observation was also used in measuring the quality of life. For example, the standardized SF-36 questionnaire is a self-reporting questionnaire measuring eight health dimensions: physical functioning, social functioning, role limitations due to physical problems, role limitations due to emotional problems, mental health, energy and vitality, bodily pain and general perception of health, as well as two summary measures—a physical component summary scale and a mental component summary scale. The idea of the study also rests on the goals of the UN World Programme of Action Concerning Disabled Persons. Moreover, since the group of people with disabilities who practice diving was relatively small, we decided against breaking it down by the degree of disability or gender in our analyses.

A study concerning scuba diving involved multiple challenges. Extensive investigation into the field requires comprehensive knowledge and experience from various scientific disciplines. When analyzing the list of references, it appears that studies on diving of healthy and fully abled individuals are easy to find. However, the scientific database concerning the diving of people with disabilities is definitely smaller. The number of papers focusing on this group is limited. It may be due to the fact that the interest in scuba diving in both the disabled themselves and in the world of science has increased only in recent years. It can also be seen in the number of divers who participated in the study (64 people) as compared to non-divers (*n* = 118). The groups of divers examined in studies carried out by other authors were also small [9,20,26,27].

Other problems (in Poland) include the high costs of diving. This is a serious limitation to wider participation in scuba diving activities for less wealthy individuals. One may seek help from NGOs (associations and foundations that provide rehabilitation services to the disabled), which, apart from raising funds, often act as a go-between in the organization of diving training and trips for their beneficiaries who cannot afford such expenses on their own.

On top of that, in Poland, studies on diving of the disabled encounter many difficulties. The reason is the little need of people with disabilities to take part in scientific research. Apparently, individuals with disabilities are not aware of the fact that the results of such studies could improve the quality of their own lives. Additionally, only a handful of Polish diving instructors have the general and specialist knowledge that allows them to conduct such classes in a professional manner.

The biggest group in the present study comprised people suffering from motoric dysfunctions, which were the main reason for the disabilities. In the majority of published studies concerning the diving of people with disabilities, the studied group was also composed of people whose disability resulted from dysfunctions of the locomotor system, such as paraplegic divers [7,26], war veterans [6,27] or people after limb amputations [20].

Due to the scuba diving rules, the need to use specialist equipment and a different environment, diving poses multiple physical and mental challenges to divers, which, as a result, contribute to the improvement of the physical and mental conditions of the disabled. The studies by Williamson et al. [26] already showed that scuba diving may improve the physical and mental conditions of the disabled. The authors studied a group of 16 people with various dysfunctions (paraplegia, head injuries and after limb amputations). This study also showed that diving may improve both the physical and mental conditions. Such was the opinion of both groups of respondents. With regards to the improvement of the physical condition, score 4 dominated; as regards the mental condition, the prevalent score was 5 (on a 5-point scale).

The studies showed that diving may improve the self-assessment of a disabled person. Cheng and Diamond [24] argued that positive experiences during diving translate into positive self-perception. They showed that, after a three-week diving program, disabled individuals (after limb amputation) had higher levels of self-assessment, and the levels of depression were significantly lower compared to the initial values. In our study, the diving respondents also noticed improvements in their self-assessments, which translated into high scores given to this parameter. The opinions of non-divers were similar.

In a study involving war veterans, Morgan et al. [27] demonstrated that participation in diving classes may help to reduce anxiety and depression levels, as well as improve social functioning and, following this, relationships with the social environment. Aganović [20] reported that, after a 3-week diving training program, the disabled individuals were well-prepared for recreational diving, comparable to fully abled people. Therefore, in the author’s opinion, a disability is not a barrier in recreational participation in that physical activity. This study also showed that diving may contribute to the improvement of social interactions. Carin-Levy and Jones [9] were of a similar opinion, and they underlined the mental and social benefits of diving. They demonstrated the improvement of self-awareness of the disabled, as well as the increase in their social experiences. They also emphasized that, in the opinion of the respondents, activities in the aquatic environment give the feeling of lightness and being free from their disability. Thus, it helps one feel equal to fully abled divers. Similarly, in our study, the respondents stated that scuba diving helped to increase their movement possibilities. It is worth emphasizing that the diving individuals scored this parameter much higher than those who did not have the opportunity to dive yet.

Classes in the aquatic environment, such as swimming and diving, engage the respiratory system more intensely. Training in breathing under water increased the peak and endurance exercise capacities in people with chronic obstructive pulmonary disease [28].

Exercises in the water improved, at a statistically significant level, the flexibility, strength and aerobic efficiency of the knees and hips in adults with a degenerative disease of the joints [29]. In the present study, we evidenced that the respondents were divided over the impact of scuba diving on the improvement of muscle strength. Both groups of respondents most often scored 4 or 3 of the Likert scale in their answers.

The aquatic environment is optimal for many rehabilitation exercises, as the loss of body weight after immersing into water enables one to make movements that are impossible or very difficult on land [12,30]. The study of Wang et al. evidenced that reduced friction movements in the water are smooth and painless, which provides great conditions for the work of weak muscles and facilitates movements, engaging painful joints or muscles [29].

Following the development of science and technology, we now have the opportunity to put into place successful strategies for preventing the exclusion of the disabled from society. Modern technologies, as well as original therapeutic strategies, provide useful tools for the rehabilitation of those with disabilities. Scuba diving for the disabled may be considered as one such solution, as it may be an alternative but efficient form of rehabilitation, both in the physical and mental aspects.

The findings of our experiments should not be generalized because of a relatively small (so far) group of disabled scuba divers. However, the descriptive analysis showed that the expectations of the people who did not dive yet on the possible benefits resulting from that activity were high. That could be seen in the scores given to the individual parameters.

Despite their limitations, individuals with disabilities may feel satisfied while scuba diving because of the support of people assisting them during diving and, also, because of the modifications of the equipment (if necessary), which would enable them to perform underwater activities. Diving is an important example of how to overcome barriers and accomplish a goal often unattainable to people without disabilities. In this case, however, the unattainable results were mostly from a lack of awareness, imagination and determination. Furthermore, diving gives people with disabilities a sense of belonging to the elite, doing something unusual, overcoming many obstacles and successfully working to improve their health status. People with disabilities prove that it is possible to exceed limits, and it is worth doing to accept one’s fate and to prove that anything is possible if you believe in it.

## 5. Conclusions

Diving may become one of the well-accepted and successful forms of rehabilitation for individuals with disabilities. It may help the disabled to improve their mental and physical conditions, which will translate into the improvement of their overall functions.

There is a need for further studies to expand the pool of knowledge on the benefits and possible health complications resulting from diving. Such studies should have a significant effect on the improvement of the quality of life of people with disabilities.

## Figures and Tables

**Table 1:** Description of the analyzed group.

Non-Divers (*n* = 118)	Category	Divers (*n* = 64)
Percent		Percent
61.8	men	73.4
38.2	women	26.6
56.6	severe degree of disability	42.2
22	moderate degree of disability	12.5
25.4	mild degree of disability	45.3
27.97	congenital disability	28.12
51.70	disabled as a result of a disease	15.63
20.33	disabled as a result of an injury	56.25
23.73	period of disability: 1–5 years	10.94
39.83	time of disability: 6–10 years	67.19
36.44	time of disability: more than 10 years	21.87
75.42	musculoskeletal system dysfunctions	82.81
18.65	dysfunctions caused by neurological diseases	10.93
5.08	vision dysfunction	3.12
0.85	hearing impairment	3.12

**Table 2:** Scuba diving impact on the mental condition.

Non-Divers		Category	Divers	
Percent	Frequency	of Likert Scale	Frequency	Percent
52.54	62	5	51	79.69
32.20	38	4	13	20.31
11.02	13	3	0	0
1.69	2	2	0	0
2.54	3	1	0	0

**Table 3:** Scuba diving impact on self-esteem.

Non-Divers		Category	Divers	
Percent	Frequency	of Likert Scale	Frequency	Percent
50.00	59	5	46	71.87
35.59	42	4	18	28.12
10.17	12	3	0	0
1.69	2	2	0	0
2.54	3	1	0	0

**Table 4:** Scuba diving impact on a belief in one’s own abilities.

Non-Divers		Category	Divers	
Percent	Frequency	of Likert Scale	Frequency	Percent
53.39	63	5	44	68.75
34.75	41	4	20	31.25
7.63	9	3	0	0
2.54	3	2	0	0
1.69	2	1	0	0

**Table 5:** Scuba diving impact on the improvement of social interactions.

Non-Divers		Category	Divers	
Percent	Frequency	of Likert Scale	Frequency	Percent
47.46	56	5	39	60.94
33.90	40	4	14	21.87
13.56	16	3	11	17.19
2.54	3	2	0	0
2.54	3	1	0	0

**Table 6:** Scuba diving impact on overall physical fitness.

Non-Divers		Category	Divers	
Percent	Frequency	of Likert Scale	Frequency	Percent
37.29	44	5	19	29.69
46.61	55	4	35	54.69
11.02	13	3	10	15.62
2.54	3	2	0	0
2.54	3	1	0	0

**Table 7:** Scuba diving impact on the improvement of muscle strength.

Non-Divers		Category	Divers	
Percent	Frequency	of Likert Scale	Frequency	Percent
8.47	10	5	3	4.69
43.22	51	4	29	45.31
30.51	36	3	28	43.75
15.25	18	2	4	6.25
2.54	3	1	0	0

**Table 8:** Scuba diving impact on the improvement of the movement ability.

Non-Divers		Category	Divers	
Percent	Frequency	of Likert Scale	Frequency	Percent
52.54	62	5	40	62.50
35.59	42	4	24	37.50
8.47	10	3	0	0
1.69	2	2	0	0
1.69	2	1	0	0

**Table 9:** Scuba diving impact on the efficiency respiratory system.

Non-Divers		Category	Divers	
Percent	Frequency	of Likert Scale	Frequency	Percent
56.78	67	5	34	53.12
12.71	15	4	12	18.75
25.42	30	3	18	28.12
3.39	4	2	0	0
1.69	2	1	0	0

**Table 10:** Scuba diving impact on pain relief.

Non-Divers		Category	Divers	
Percent	Frequency	of Likert Scale	Frequency	Percent
9.32	11	5	3	4.68
74.58	88	4	46	71.87
10.17	12	3	13	20.31
3.39	4	2	2	3.12
2.54	3	1	0	0

## Data Availability

Data is contained within this article.
